# Rarely Encountered Gram-Negative Rods and Lung Transplant Recipients: A Narrative Review

**DOI:** 10.3390/microorganisms11061468

**Published:** 2023-05-31

**Authors:** Eric Farfour, Antoine Roux, Edouard Sage, Hélène Revillet, Marc Vasse, Alexandre Vallée

**Affiliations:** 1 Service de Biologie Clinique, Hôpital Foch, 92150 Suresnes, France; 2 Service de Pneumologie et Transplantation Pulmonaire, Hôpital Foch, 92150 Suresnes, France; 3 Service de Chirurgie Thoracique et Transplantation Pulmonaire, Hôpital Foch, 92150 Suresnes, France; 4 Service de Bactériologie-Hygiène Hospitalière, CHU de Toulouse, 31300 Toulouse, France; 5 Observatoire National *Burkholderia cepacia*, 31403 Toulouse, France; 6 INSERM Hémostase Inflammation Thrombose HITH U1176, Université Paris-Saclay, 94276 Le Kremlin-Bicêtre, France; 7 Service d’Epidémiologie-Data-Biostatistiques, Délégation à la Recherche Clinique et à l’Innovation, Hôpital Foch, 92150 Suresnes, France

**Keywords:** acetobacter, bordetella, chryseobacterium, elizabethkinga, inquilinus, pandoraea, lung transplant recipient

## Abstract

The respiratory tract of lung transplant recipients (LTR) is likely to be colonized with non-fermentative Gram-negative rods. As a consequence of the improvements in molecular sequencing and taxonomy, an increasing number of bacterial species have been described. We performed a review of the literature of bacterial infections in LTR involving non-fermentative Gram-negative rods with exclusion of *Pseudomonas aeruginosa*, *Stenotrophomonas maltophilia*, *Achromobacter* spp. and *Burkholderia* spp. Overall, non-fermenting GNR were recovered from 17 LTR involving the following genera: *Acetobacter*, *Bordetella*, *Chryseobacterium*, *Elizabethkinga*, *Inquilinus*, and *Pandoraea*. We then discuss the issues raised by these bacteria, including detection and identification, antimicrobial resistance, pathogenesis, and cross-transmission.

## 1. Introduction

The respiratory tracts of patients with chronic lung disease and lung transplant recipients (LTR) is likely to be colonized with non-fermentative Gram-negative rods (GNR) such as *Pseudomonas aeruginosa*, *Stenotrophomonas maltophilia*, *Achromobacter* spp. or *Burkholderia* spp., the latter being mainly recovered from cystic fibrosis (CF) patients. Since the beginning of the 2000s, improvements in molecular sequencing and bacterial taxonomy have led to an increasing number of bacterial genus and species description. Bacterial families of closely related species of well described pathogens were extended: *Alcaligenaceae* (e.g., *Achromobacter* spp.), *Burkholderiaceae* (e.g., *Burkholderia* spp.), *Pseudomonadaceae* (e.g., *P. aeruginosa*), *Xanthomonadaceae* (e.g., *S. maltophilia*). Furthermore, bacterial genera were reclassified as members of the families *Rhodospirillaceae*, *Flavobacteriaceae*, *Acetobacteraceae*, and *Weeksellaceae.* Some species belonging to these families were associated with infections in immunocompromised patients or patients with chronic respiratory diseases such as CF. Most of these bacterial species are environmental saprophytes that can be recovered from soil, plants, or water. They present characteristics that raise questions and challenges: (i) as they are rarely isolated, they could be under-recognized as pathogens; (ii) their detection and identification in clinical laboratories could be problematic; (iii) multidrug-resistance is common and specific guidelines are lacking to assess their susceptibility to antimicrobial drugs; (iv) colonization of the respiratory tract and infection might be difficult to distinguish, (v) finally, hospital cross-transmission and its prevention remains unclear.

The incidence of post-transplant infections is higher in LTR than in other solid-organ recipients [1]. Most infections are due to common respiratory viruses such as influenza. However, bacterial infections are not uncommon. Early post-transplant infections mainly involve the bacteria of the recipient rather than those of the donor [2]. Consequently, LTR previously colonized with non-fermenting Gram-negative rods (GNR) are at risk of infection involving these bacteria. Conversely, late-onset infections could involve a wide range of bacteria including vaccine-preventable pathogens such as *Streptococcus pneumoniae* or *Haemophilus influenzae*.

We performed a narrative review of bacterial infections in LTR involving uncommon species of the families Burkholderiaceae, Rhodospirillaceae, Pseudomonadaceae, Weeksellaceae, Flavobacteriaceae, Alcaligenaceae, Acebobacteraceae, and Xanthomonadaceae. We retrieved published cases of uncommon GNR recovered from LTR to identify singularities related to their, (i) detection and identification, (ii) antimicrobial resistance, (iii) role in infections, and (iv) cross-transmission and prevention.

## 2. Methods of the Narrative Review

**Figure 1:**
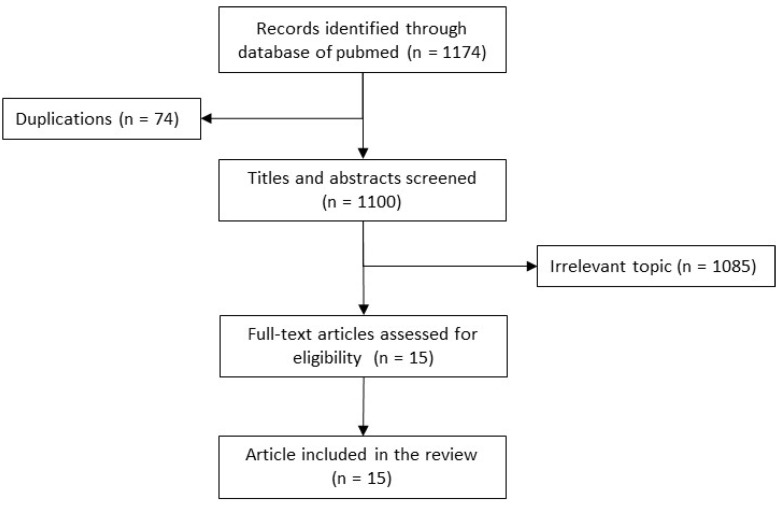
Flow diagram. Relevant article Detection, Identification, Antimicrobial resistance, cross-transmission and its prevention.

Studies were searched from PubMed from inception to 30 September 2022. All bacterial genera belonging to 8 bacterial families *Acetobacteraceae*, *Alcaligenaceae*, *Burkholderiaceae*, *Flavobacteriaceae*, *Pseudomonaceae*, *Rhodospirillaceae*, *Xanthomonadaceae*, *Weeksellaceae* as defined by the Taxonomy browser of the National Library of Medicine were included in the general review [3]. 1174 articles were identified from PubMed of which 15 case reports were found eligible (Figure 1).

The common species and complex *Pseudomonas aeruginosa*, *Achromobacter xylosoxydans*, *Burkholderia cepacia* complex, and *Stenotrophomonas maltophilia* were excluded. Case reports and cohorts of LTR from whom non-fermenting GNR were isolated whether the patients were infected or not were eligible for review. Considering case-reports and case-series, the following data were extracted from each eligible article: socio-demographic characteristics, the reason for lung transplantation, other predisposing conditions, past medical history including antimicrobial treatment prior to bacterial isolation, clinical findings at the time of isolation and associated micro-organisms, and outcome. Data relative to detection and identification, antimicrobial resistance, role in infection, cross-transmission and its prevention were extracted. Overall, non-fermenting GNR were recovered from 17 LTR involving the following genera: *Acetobacter*, *Bordetella*, *Chryseobacterium*, *Elizabethkinga*, *Inquilinus*, and *Pandoraea* (Table 1). Except for the genera *Bordetella*, all could be classified as opportunistic environmental saprophytes.

## 3. Review of Cases (Table 2)

### 3.1. Acetobacter indonesiensis

Two cases of *A. indonesiensis* infections have been reported in LTR over the past years [4,5]. One of them was a 31-year-old man with CF and the second was a 51-year-old woman with hypersensitivity pneumonitis, extrinsic allergic alveolitis, and short telomere syndrome. Neither of them was previously known to be colonized with *A. indonesiensis*. In both patients, *A. indonesiensis* was recovered from at least three respiratory samples with the first positive one within the month following the lung transplantation. *A. indonesiensis* was not recovered from other samples such as blood cultures. Despite both patients being considered infected, they recovered with antibiotic drugs inactive against *A. indonesiensis. A. indonesiensis* was not recovered in any subsequent respiratory tract sample after seven months of follow-up for one of them.

### 3.2. Chryseobacterium *spp.*

*Chryseobacterium* spp. was mentioned once in a retrospective series of clinically relevant infections following lung transplantation in South Korea [6]. The patient developed pneumonia between 1 and 6 months following the transplantation. No other microbiological or clinical data are available for this case.

### 3.3. Elizabethkinga *spp.*

*Elizabethkinga* spp. was associated with septic shock in a 26 year-old man who had received lung transplantation five years before for end-stage CF [7]. The patient was first admitted for hypoxic respiratory failure attributed to the respiratory syncytial virus. During his hospital stay, he developed septic shock. Elizabethkinga was isolated from blood culture, peritoneal fluid, and bronchoalveolar lavage (BAL). Microbiological clearance was obtained with intravenous piperacillin-tazobactam and levofloxacin. However, on day 15, he developed a cardiac arrest secondary to acidosis and severe hypercapnic respiratory failure. He died on day 19 of his hospital stay.

### 3.4. Inquilinus limosus

*I. limosus* was isolated from 3 LTR with early post-operative infections [8,9,10]. All of them were transplanted for CF and were previously colonized with *I. limosus*. The first one was a 22-year-old woman who presented with pulmonary infiltrate one week after lung transplantation [8]. *I. limosus* was isolated with *Enterococcus* spp. and coagulase-negative *Staphylococcus* from a BAL. The patient recovered and was discharged 6 weeks after the transplantation. *I. limosus* was not isolated from her respiratory tract after one year of follow-up. The second patient, a 31-year-old man, successively developed a bacteraemic lung empyema on post-operative day 38, and a contralateral lung empyema 8 months later [9]. *I. limosus* was recovered from blood culture and the empyema during the first infection and the empyema only during the second one. He completely recovered from both episodes with surgical and antimicrobial treatment. The authors did not report whether *I. limosus* was isolated from the patient between the two infections and after the second one. The last patient, a 45-year-old LTR woman eight years ago, presented an *I. limosus* bacteraemia after a SARS-CoV-2 infection [10]. *I. limosus* was isolated from three consecutive respiratory samples and from an aerobic blood culture vial. She recovered with an antimicrobial regimen. She was colonized before the lung transplantation, but *I. limosus* has not been isolated since then.

### 3.5. Pandoraea *spp.*

*Pandoraea* spp. was the most frequent bacterial genera isolated from LTR with six patients infected. Five patients were lung transplanted for CF, while the remaining one was a 30-year-old man with end-stage pulmonary sarcoidosis complicated by nocardiosis and mycetomas [11]. *P. pnomenusa* was never isolated from this latter patient before the lung transplantation. Immediately post-operatively he presented with sepsis and pulmonary effusions. *P. pnomenusa* was isolated from multiple sets of blood cultures and respiratory samples. No other pathogens were found. The patient developed progressive acute respiratory distress syndrome and died on post-operative day 17 from refractive septic shock and multiple organ failure.

All five lung transplanted patients for CF were previously colonized with *Pandoraea* spp. Post-operatively, *Pandoraea* spp. was associated with colonization in three patients and infection in two patients. One patient was a 30-year-old female who died 3 weeks after lung transplantation from *Pseudomonas aeruginosa* septic shock [12]. The two other patients were doing well after lung transplants [13,14]. Of the two infected patients, a 21-year-old woman died from *P. noserga* septic shock on post-operative day 25 [15]. The second patient, a 30-year-old woman, developed a pleural effusion positive for *P. apista* and *P. aeruginosa* on post-operative day 45 [14]. She recovered with appropriate treatment.

Of note, one CF patient colonized with an isolate of *P. sputorum* was recused for lung transplantation due to concern about the isolate [16]. However, *P. apista* clearance was reported following lung transplantation in a 21 year-old woman with CF [17].

### 3.6. Bordetella *spp.*

In contrast to the other genus for which an environmental source is suspected or proved, *Bordetella* species are mainly transmitted through contact with infected humans or pets. Furthermore, while *B. pertussis* and *B. parapertussis* are fastidious micro-organisms that cultivate in vitro on Bordet-Jengou selective agar, other *Bordetella* species such as *B. bronchiseptica*, *B. petrii*, *B. trematum* grow on standard media such as blood Columbia or chocolate agar.

A single case of *B. pertussis* infection was reported in an LTR for CF [18]. The patient presented with acute respiratory distress syndrome five years after the transplantation. He recovered from whooping cough with antibiotics, but he required a prolonged ICU stay complicated by hypoxic-ischaemic optic neuropathy leading to blindness. His sister tested positive for *B. pertussis* during his hospital stay. Nevertheless, the author did not report if the patient had been vaccinated against whooping cough or not.

Of the three patients with *B. bronchiseptica* infection, two were transplanted for CF and one for anti-MDA-5-associated clinically amyopathic dermatomyositis [19,20]. They were all in contact with dogs and two contracted a Kennel cough within six months following the transplantation, while the third contracted it one year later. Vaccination status of the contact pet is reported in a single case, a puppy was not vaccinated against *B. bronchiseptica* [19]. All three patients required hospital management of *B. bronchiseptica* infection. Two of them recovered with an appropriate antibiotic regimen, while a 10-year-old girl died from respiratory failure.

**Table 2:** Cases involving opportunistic Gram-negative rods.

No.	Age	Sex	Reason for Lung Transplantation	Infection	Isolation before Transplant	Species	Other Associated Pathogens	Past History of Antimicrobial Treatment	Medical History	Outcome	Ref.
1	31	M	Cystic fibrosis	Early post-operative pneumonia/Colonization	No	*A. indonesiensis*	*P. aeruginosa* *S. aureus*	Peri-operative prophylaxis with colistin, tobramycin, ceftazidime, and linezolid	Three sputa sampled on post-operative day 11, 21, and 30 positive for *A. indonesiensis* Patient improved despite ineffective antimicrobial drug against *A. indonesiensis* *A. indonesiensis* was not recovered during 7 months of follow-up	Recovery	[21]
2	51	F	- Hypersensitivity pneumonitis, - Extrinsic allergic alveolitis - Short telomere syndrome	Early post-operative Pneumonia/Colonization	No	*A. indonesiensis*	n.a. *	Post-operative prophylaxis cotrimoxazole, vancomycin, piperacillin/tazobactam	Three post-operative respiratory samples (1 tracheostomy suction and 2 BAL **) positive for *A. indonesiensis* from post-operative day 21. Patient improved despite ineffective antimicrobial drug against *A. indonesiensis.* *A. indonesiensis* was not recovered during follow-up	Recovery	[4]
3	n.a.	n.a.	n.a.	Pneumonia 1 to 6 months after transplant	n.a.	*Chryseobacterium* spp.	n.a.	n.a.	n.a.	n.a.	[6]
4	26	M	Cystic fibrosis	Septic shock 5 years after transplant	No	*Elizabethkinga* spp.	*P. aeruginosa*	n.a.	Admission for hypoxic respiratory failure attributed to respiratory syncytial virus bronchiolitis Day 6 of hospital admission: septic shock. *Elizabethkinga* spp. isolated from blood culture, peritoneal fluid and BAL. Effective antibiotic therapy with piperacillin/tazobactam and levofloxacin. Day 15: cardiac arrest secondary to acidosis and severe hypercapneic respiratory failure Day 19: death. Repeated blood and tracheal secretions cultures during hospital stay showed clearance of *Elizabethkinga* spp.	Death	[7]
5	22	F	Cystic fibrosis	Early post-operative pulmonary infiltrate (week 1)	Yes	*I. limosus*	n.a.	Peri-operative prophylaxis with imipenem, tobramycin, ceftazidime, and aerosolized colistin	On postoperative week 1: development of pulmonary infiltrate. A BAL performed on Day 8 was negative. Post-operative day 16: BAL positive for *I. limosus*. The patient improved with antibiotic regimen and was discharged from the hospital 6 weeks after the transplant *I. limosus* was not isolated within 1 year of follow-up.	Recovery	[8]
6	31	M	Cystic fibrosis	Early bacteraemic lung empyema on postoperative day 38 and month 8	Yes	*I. limosus*	no	Peri-operative prophylaxis with piperacillin-tazobactam, tobramycin, cotrimoxazole, nebulized amphotericin, azithromycin	Immediate pulmonary infiltrates after lung transplantation. Postoperative day 11. *I. limosus* was isolated from blood culture. On day 38, he presented pleural effusions, erythema of thoracotomy, ECMO wound sites, and lung empyema. He recovered with antimicrobial drugs and surgery. Seven months later he developed a contralateral lung empyema. *I. limosus* was isolated from pleural fluid. He recovered with antimicrobial drugs and surgery.	Recovery	[9]
7	45	F	Cystic fibrosis	Bacteremia 8 years after lung transplantation	Yes	*I. limosus*	No	n.a.	One year before infection, progressive graft dysfunction with no obvious trigger treated with alemtuzumab Hospital admission three weeks after COVID-19 diagnosis for worsened respiratory conditions *I. limosus* isolated from three consecutives respiratory samples and one blood culture vial. Treatment with meropenem plus amikacin switched for ciprofloxacin after identification of *I. limosus* Respiratory improvement	Recovery	[10]
8	30	F	Cystic fibrosis	Early post-operative pleural effusion	Yes	*P. apista*	*P. aeruginosa*	n.a.	Postoperative Day 0: post-transplant cultures negative for *P. apista* Post-operative day 45: pleural effusion that growth *P. apista* and *P. aeruginosa*. A bronchial wash was also positive for *P. apista*. She recovered with antimicrobial therapy. *P. apista* was never recovered after one year of follow-up.	Recovery	[14]
9	36	M	Cystic fibrosis	Colonization	Yes	*P. apista*	n.a.	n.a.	The first post-operative bronchial washing was positive for *P. apista*. The patient did not present symptoms of infection. No further samples were positive for *P. apista*.	Recovery	[14]
10	21	F	Csytic fibrosis	Early post-operative septic choc	Yes	*P. nosoerga*	No	Peri-operative prophylaxis piperacillin/tazobactam, tigecyclin	Post-operative day 5: *P. nosoerga* septic choc treated with meropenem plus tygecyclin, then meropenem plus minocyclin with initially good response. Post-operative day 14: second *P. nosoerga* septic choc. Antibiotics were switched for doxycycline + rifampincin + risperidone Replacement of ECMO canulation as a consequence of recurrent bacteremia. Post-operative day 25: death attributed to refractory spectic choc caused by *P. nosoerga*	Death	[15]
11	30	M	End-stage pulmonary sarcoidosis complicated by nocardiosis and mycetomas. Prednisone 50 mg daily	Septic shock	No	*P. pnomenusa*	n.a.	Peri-operative prophylaxis Ceftazidime, vancomycin.	Immediate post-operative shock with bilateral pleural effusions. Post-operative day 4: development of progressive bilateral pulmonary infiltrates. Post-operative day 10: *P. pnomenusa* was isolated from a BAL. The patient developed progressive respiratory distress syndrome, coagulopathy, and refractory hypotension Postoperative day 17: death attributed to refractory shock and multiple organ failure.	Death	[11]
12	n.a.	n.a.	Cystic fibrosis	Colonization	n.a.	*P. pulmonicola*	n.a.	n.a.	n.a.	Recovery	[13]
13	30	F	Cystic fibrosis	Colonization	Yes	*P. pulmonicola*	*P. aeruginosa*	n.a.	Postoperative day 5: Her condition worsened. *P. aeruginosa* was isolated from blood cultures, lymph nodes, bronchoalveolar lavage, liquid drains, and pleural liquid Postoperative day 19: *P. pulmonicola* cultured only from one BAL.A CT scan of the lung showed diffuse bilateral alveolar opacities and a parenchymal cavity 40 mm in diameter in the left upper lobe (Figure 2B). Postoperative week 3: death attributed to multi-organ failure consecutive to a septic shock.	Death	[12]
14	42	M	Cystic fibrosis	ARDS 5 years post-transplant	no	*B. pertussis*	No	n.a.	The patient was admitted to the intensive care unit (ICU) with fever, flu-like symptoms, and acute hypoxaemic respiratory failure. Vancomycin, piperacillin—tazobactum, azithromycin, and voriconazole was initiated. Levofloxacin was added. He underwent a tracheostomy on day 27 and was liberated from mechanical ventilation on day 64	Recovery	[18]
15	10	F	Cystic fibrosis	Late post-transplant pneumonia (1-year)	no	*B. bronchiseptica*	*P. aeruginosa*, *A. fumigatus*, *K. pneumoniae*, *S. maltophilia*.	n.a.	The patient first presented with an increased cough and a right lower lobe atelectasis/infiltrate on chest radiograph. Culture of BAL grew *P. aeruginosa*, *A. fumigatus*, *K. pneumoniae*, and *S. maltophilia.* She was started with Intravenous tobramycin and meropenem, oral cotrimoxazole, and clarithromycin that were switched for oral ciprofloxacin on discharge as she improved. She was readmitted 2 weeks later for increased cough, pleuritic chest pain, persistent right lower lobe atelectasy. *B. bronchiseptica*, *P. aeruginosa* were isolated from her BAL. She was treated with multiple courses of antibiotics including meropenem, tobramycin and ciprofloxacin, doxycycline, and piperacillin/tazobactam. Her condition continued to deteriorate, and she died due to respiratory failure	Death	[19]
16	15	M	Cystic fibrosis	Bronchitis 3 months post-transplant	no	*B. bronchiseptica*	*B. cepacia*	n.a.	Bronchitis 3-months after lung transplantation. The patient recovered with oral ciprofloxacin. Repeat sputa sampled 4 weeks later was negative for *B. bronchiseptica*.	Recovery	[19]
17	51	M	MDA-5 associated clinically amyopathic dermatomyositis.	Bronchitis 4 months post-transplant	no	*B. bronchiseptica*	No	n.a.	The patient presented with onset cough four months after lung transplant. *B. bronchiseptica* was isolated from BAL. He recovered with oral ciprofloxacin and inhaled amikacin.	Recovery	[20]

* n.a.: not available. ** BAL: Broncho-alveolar fluid.

## 4. Issues Raised by Opportunistic GNR

### 4.1. Detection and Identification

These fastidious non-fermenting Gram-negative rods present several microbiological characteristics that make their culture, identification, and antimicrobial susceptibility testing (AST) challenging in clinical laboratories. The results of external quality control trials of diagnostic microbiology of CF isolates highlight these difficulties [22]. A total of 31 and 37 laboratories that handled CF patient samples participated in this program in 2008 and 2009, respectively [22]. While *P. aeruginosa*, *Staphylococcus aureus*, *Burkholderia* spp. strains were correctly recovered and identified by most participants, 19.4% and 45.2% did not detect or misidentify a strain of *P. pnomenusa*, respectively. These rates were 27.0% and 10.8%, respectively for a strain of *I. limosus*. However, since these programs, methods of identification have been improved and the diffusion of the MALDI-TOF mass-spectrometry should have led to a decrease in the rate of misidentification.

Respiratory samples are frequently polymicrobial. Consequently, slow-growing micro-organisms such as *I. limosus* or *Pandoraea* spp. could be overgrown by bacteria of the commensal flora, such as staphylococci, streptococci, *Capnocytophaga* spp., or fast-growing pathogens such as *P. aeruginosa*. However, they could be cultivated on a *Burkholderia cepacia* selective agar plate that could therefore enhance their recovery and their isolation. However, *Burkholderia* selective agar is mainly used for respiratory samples of CF patients but not those from lung transplant recipients or patients with other respiratory diseases [23]. The respiratory samples of CF patients are incubated for 5 days to allow the growth of slow-growing micro-organisms. Indeed, among the 17 patients included in the present review, 14 were for CF with sputa and were incubated for 5 days. While bronchoscopy samples are incubated for 4 or 5 days, sputa from LTR are incubated only for 2 days, and consequently do not allow the culture of fastidious GNR. The benefit of the use of *Burkholderia* selective agar media and the prolonged incubation of cultures from LTR, as for CF samples, remains to be assessed. Furthermore, the morphotype of the colonies is not specific and it is sometimes confusing. For instance, *I. limosus* colonies display a mucoid morphotype similar to mucous strains or *P. aeruginosa*. Consequently, they are likely misidentified as this pathogen using morphologic characteristics only [24].

Phenotypic and biochemical methods provide frequently inconsistent identification of these species, i.e., either no identification or misidentification [16,25,26,27,28,29]. Indeed, *Ralstonia* spp. were previously misidentified as *Burkholderia* spp., *I. limosus* as *Roseomonas gilardii*, *Sphingomonas paucimobilis* or *Agrobacterium radiobacter* [30,31,32]. Conversely, some strains were misidentified as belonging to these species. For instance, a *Burkholderia gladioli* strain has been misidentified as *Empedobacter* spp, a genus of the *Weeksellaceae* family [33], and *B. trematum* could be misidentified as *B. bronchiseptica* [34]. These lower performances could be related to inherent limitations of biochemical methods [35]: (i) their databases are limited and could lack several species; (ii) not all of the strains of the same species have the same characteristics; (iii) the same strain may produce different results in repeated tests. MALDI-TOF mass spectrometry has greatly improved the identification of cultivable micro-organisms in clinical laboratories including fastidious bacteria [36,37]. In comparison to 16S rDNA gene sequencing, most of the clinical isolates are correctly identified at the species level [38,39]. Furthermore, MALDI-TOF mass spectrometer databases are regularly updated to include new species and improve the identification of closely related species that remain difficult to distinguish. However, closely related species, for instance, *Elizabethkingia* species, could not be distinguished using a MALDI-TOF mass spectrometer equipped with commercial reference databases [40]. In-house expanded spectrum databases could be implemented for their identification [41]. The 16S rDNA gene sequencing displays high performance for the identification of all these bacteria. However, it is not able to distinguish some closely related species that display a high level of sequence homology [31,42]. Alternative molecular methods such as *gyrB* gene sequencing or specific PCR could resolve ambiguous identifications but they required specific reagents and skills. Consequently, they are performed by a low number of laboratories [21,43,44,45,46,47].

### 4.2. Antimicrobial Resistance

Assessing the antimicrobial susceptibility of opportunistic GNR is also challenging. Species-related methods (inoculum, temperature, and delay of incubation) and breakpoints lack in both CLSI and EUCAST guidelines [48,49,50]. Antimicrobial susceptibility testing was mainly performed and interpreted using *Burkholderia* spp., *Pseudomonas aeruginosa*, or non-related species guidelines. As they are slow-growing the incubation could require a prolonged delay of up to 48 h.

Species’ intrinsic antimicrobial resistance remains unclear. Most data are from case reports or case series that included a few strains. Furthermore, a high rate of strains was recovered from CF patients that could have been colonized for several years or treated with several courses of antimicrobials. Consequently, the pattern of wild-type strains is little assessed. Considering these findings, performing AST is therefore mandatory when an antibiotic regimen is required. In colonized patients, antimicrobial susceptibility of previously recovered strains should be considered when empiric therapy is required.

In vitro, most β-lactams including β-lactams/β-lactamases inhibitor combinations are inactive except for the carbapenems [4,5,25,26,51,52,53,54,55]. However, the emergence of meropenem resistance in an *I. limosus* strain was reported during a meropenem course in a 16 year-old girl with CF [24]. Fluoroquinolones, aminoglycosides, and cyclins exhibit a variable activity [4,5,25,26,51,52,53,54,55,56]. Colistin is inactive in vitro against *Inquilinus* spp., *Pandoraea* spp., *Ralstonia* spp., and *A. indonesensis* [4,5,25,51,52,54,55]. It has been suggested that the clinical use of nebulized colistin to treat *P. aeruginosa* infection in CF patients enhances the selection and colonization of colistin-resistant species [21]. Fosfomycin and trimethoprime-sulfametoxazole combination were almost always found inactive [25,51,52,54], suggesting in a comparable manner to other non-fermenting Gram-negative rods, the drugs are inactive in vivo against these species.

Genetic determinants of resistance have been identified mainly for β-lactams. All these species intrinsically express broad-spectrum β-lactamases such as OXA-62 in *Pandoraea* spp. [57,58], OXA-22 and OXA-60 in *Ralstonia* spp. [59], Inq1 in *I. limosus* [56], BlaB and GOB in *Elizabethkingia* spp. [60,61], or IND-16 and OXA-209 *Chryseobacterium* spp. [62,63,64]. Some strains were found to harbour several genes coding β-lactamases [63,65]. However, broad-spectrum β-lactamases genes could be either chromosomally or plasmid-encoded, *Elizabethkingia* spp. has the singularity of harbouring a high diversity of chromosomally encoded Metallo-β-lactamases [66]. Moreover, other mechanisms including efflux-pump, genomic mutation, or methylation could take part in antimicrobial resistance to β-lactams and other classes of drugs such as fluoroquinolones [42,67,68,69].

### 4.3. Pathogenesis

All these opportunistic GNR could colonize the respiratory tract of patients with underlying diseases. *I. limosus* and *Pandoraea* spp. were almost exclusively recovered from the respiratory tract of CF patients [14,21,24]. Conversely, *Chryseobacterium* spp. and *Elizabethkinga* spp. have been isolated from various clinical contexts including meningitis in newborns or bacteremia in immunocompromised patients [40,70,71,72,73,74].

Interestingly, the two patients with *I. limosus* early post-operative infections were previously colonized and both recovered from their infection. *I. limosus* colonization in CF patients is associated with specific serum antibody response [24]. It can be speculated that the antibodies against *I. limosus* could have contributed to favorable outcomes in these patients. Of the four patients previously colonized with *Pandoraea* spp. prior to lung transplantation, two were found to have been colonized after the surgery, one recovered from *P. apista* septic shock, and one died from *P. pnomenusa* septic shock. The potential role of immune response consecutive to chronic colonization remains to be assessed for this genus.

Considering *A. indonesiensis*, despite the bacteria being recovered from multiple respiratory samples in both patients [4,5], colonization rather than infection could not be ruled out as both recovered without effective antimicrobials.

Indeed, as they could be associated with chronic colonization of the respiratory tract, the significance of their recovery remains unclear. However, clinical signs, spirometry, and declining radiographic parameters were reported in some patients following the isolation of *I. limosus* and *Pandoraea* spp. [24,75,76,77]. The outcome of CF patients colonized with *Inquilinus* spp was assessed in a retrospective case-control study that included 17 patients with at least one respiratory culture positive for *Inquilinus* spp compared with age-matched CF controls with chronic *P. aeruginosa* colonization [53]. After five years of follow-up, the patients colonized with *I. limosus* showed a similar decrease in spirometry value, BMI percentiles, and rate of pulmonary exacerbation, to that of those colonized with *P. aeruginosa*.

In silico analyses have suggested their genomes encoded several virulence factors and determinants associated with environmental survival and encompassed genes associated with flagella, adhesion, capsule polysaccharide synthesis and antiphagocytosis, secretion systems, cytokine production and cytotoxicity, and biofilm formation for instance [63,67,78,79]. The genome of *Elizabethkingia* is predicted to encode 270 putative virulence factors [80]. While different species of *Elizabethkingia* shared the same virulence factors, 162 predicted genes for virulence factors have been reported as unique in *Elizabethkingia anophelis* and 6 in *Elizabethkingia meningoseptica*.

*Inquilinus* spp. strain LMG 20,952 produces two exopolysaccharides (EPS) that exhibit the same charge per sugar residue as alginate, an exopolysaccharide produced by *P. aeruginosa* [81,82]. Alginate is involved in biofilm formation and also enhances adhesion to solid surfaces. However, *Inquilinus* spp. EPS displays a different confirmation suggesting it could have a specific role [83]. *Pandoraea* spp. strains were able in vitro to invade lung epithelial cells and kill *Galleria mellonella* larvae [13,84]. Virulence was inconsistent regarding bacterial strain and species. However, *P. pulmonicola* strains were generally the most virulent of the species tested in comparison to other species, *P. apista* and *P. pnomenusa*, or to *Burkholderia cenocepacia*. *Chryseobacterium* spp. and *Elizabethkingia* spp. are able in vitro to produce biofilm, the latter could invade respiratory tract epithelial cells in a murine model [85,86,87,88].

The airways are polymicrobial and bacteria have mutual interactions [89,90]. The kinetics of biofilm formation by *I. limosus* is disturbed in the presence of *P. aeruginosa* [91]. However, bacterial growth in a polymicrobial environment protects the target microorganism from the effect of the antimicrobial agent [91,92].

### 4.4. Cross-Transmission and Prevention

While there are no specific guidelines for *Pandoraea* spp., *I. limosus*, and *Ralstonia* spp., CF patients have been previously placed on isolation procedures during hospitalization when colonized or infected with these micro-organisms [12,14,93,94]. Furthermore, in a CF centre, an epidemic spread of *P. pulmonicola* was controlled by implementing additional cross-transmission prevention measures [95]. Of note, preventative measures were not strictly followed in the CF department before additional control measures were implemented.

It was suggested that arrangements must be set up for CF patients infected with *Burkholderia cepacia* complex and methicillin-resistant *Staphylococcus aureus*, for example, separate clinics and appropriate inpatient segregation [96,97]. However, the American guidelines consider the lack of definitive support for cohort segregation. Nevertheless, people with CF should be separated from other patients regardless of their respiratory tract culture results [98]. In CF patients, additional infection prevention and control (IPC) measures are controversial as they are mainly based on theoretical benefit more than proven efficacy [99].

Regarding the low frequency of isolation of these fastidious GNRs and the absence of evidence in LTR, specific infection prevention and control measures are probably not required. The compliance with standard precautions, which is inconsistent in several wards including CF centers [100,101], should be enhanced in order to prevent the cross-transmission of all microorganisms from asymptomatic and symptomatic carriers. Indeed, to be successful, infection control measures need to be simple, universally applied, and acceptable [99]. In addition, as for CF, LTR should probably be separated from other patients regardless of their respiratory tract culture results.

## 5. Issues Raised by *Bordetella* spp.

In contrast to the other genus for which an environmental source is suspected or proved, *Bordetella* species are mainly transmitted through contact with infected humans or pets. While they belong to the same genus, *Bordetella* species differ in several characteristics. *B. pertussis* and *B. parapertussis* are fastidious micro-organisms that cultivate in vitro on selective agar such as Bordet-Jengou media. Due to the lower prevalence of whooping cough and the development of PCR few laboratories now perform *B. pertussis* and *B. parapertussis* culture. Commercial or in-house PCR assays are also available for these pathogens. Consequently, clinical laboratories should be aware when *B. pertussis* and *B. parapertussis* are suspected. Conversely, other *Bordetella* species such as *B. bronchiseptica*, *B. petrii*, and *B. trematum* grow on standard media such as Columbia supplemented with blood or chocolate agar.

*B. bronchiseptica* is a zoonotic respiratory infectious agent of Kennel cough. The bacteria is transmitted by companion animals, mainly dogs and cats. The prevalence of *B. bronchiseptica* was assessed to be up to 4.9% in symptomatic cats and up to 19.5% in rescue catteries [102,103,104]. Bacterial isolation from healthy dogs ranged from 0.0% to 45.6% while it ranged from 3.3% to 78.7% in dogs with respiratory disease [105]. To date, no human transmission of *B. bronchiseptica* has been reported. *B. bronchiseptica* is mainly associated with bronchitis and pneumonia in immunocompromised patients or those with chronic lung disease.

Whooping cough and Kennel cough are preventable infectious diseases. For whooping cough, the vaccine status of the only case included in the present review was not available. While the vaccine response of LTR could differ from immunocompetent patients, there are no specific guidelines for LTR. The Pertussis vaccine is recommended or mandatory for newborns and infants in several European countries [106]. However, vaccine schedules differ among countries [106]. When possible, vaccination should be started already during the pre-transplantation period when the patient is on the waiting list. Booster vaccinations should be given post-transplantation, but only when immunosuppression has been tapered. Parental vaccine cocooning prevents pertussis infection in infants [107,108]. A similar strategy is also probably adapted for close contact with LTR. Consequently, we suggest recommendations for Pertussis vaccination should be encouraged in LTR as well as persons of close environment. Assessing immune response to pertussis vaccine in LTR is required.

We found little data regarding the vaccination of contact pets of LTR. Of the three LTR infected with *B. bronchiseptica*, the contact pet was vaccinated in a single case. Nevertheless, as an effective vaccine is available for dogs [109], LTR should be encouraged to vaccinate companion animals. Furthermore, the pertussis vaccine offers some cross-protection against *B. bronchiseptica* and therefore helps further mitigate the risk of zoonotic infection of this organism from pets to their owners [110]. While CF patients are aware of the risk of Whooping cough and the benefit of vaccination [109], a similar campaign should be in place for patients with other chronic lung diseases.

## 6. Conclusions

There are few reports of bacterial infections involving uncommon GNR in LTR, and their incidence remains to be assessed. Several methods could enhance the recovery of these bacteria, especially from polymicrobial samples such as respiratory tract samples. The use of selective media such a *Burkholderia cepacia* selective agar and prolonged incubation for the recovery of fastidious microorganisms should probably be assessed. Antimicrobial susceptibility testing and interpretation should also be standardized as well as assessing the intrinsic species pattern of resistance. Whooping cough and kennel cough are preventable infectious diseases. LTR and their relations should be encouraged to be vaccinated as well as companion animals for *B. bronchiseptica.*

## Figures and Tables

**Table 1:** Bacterial genera involved in infections or colonization in lung transplant recipients.

Family	Genera (Number of Report)
Acebobacteraceae	Acetobacter (2)
Alcaligenaceae	Achromobacter *, Bordetella (4)
Burkholderiacaea	Burkholderia *, Pandoraea (6)
Flavobacteriaceae	None
Pseudomonaceae	Pseudomonas *
Rhodospirillaceae	Inquilinus (3)
Xanthomonadaceae	Stenotrophomonas *
Weeksellaceae	Chryseobacterium (1), Elizabethkinga (1)

* Excluded from the present review.

## Data Availability

Not applicable.
